# Disulfide Linkages in *Plasmodium falciparum* Plasmepsin-I Are Essential Elements for Its Processing Activity and Multi-Milligram Recombinant Production Yield

**DOI:** 10.1371/journal.pone.0089424

**Published:** 2014-02-20

**Authors:** Sirisak Lolupiman, Pilaiwan Siripurkpong, Jirundon Yuvaniyama

**Affiliations:** 1 Department of Biochemistry and Center for Excellence in Protein Structure and Function, Faculty of Science, Mahidol University, Bangkok, Thailand; 2 Department of Medical Technology, Faculty of Allied Health Sciences, Thammasat University, Pathumthani, Thailand; RWTH Aachen University, Germany

## Abstract

*Plasmodium falciparum* plasmepsin-I (PM-I) has been considered a potential drug target for the parasite that causes fatal malaria in human. Determination of PM-I structures for rational design of its inhibitors is hindered by the difficulty in obtaining large quantity of soluble enzyme. Nearly all attempts for its heterologous expression in *Escherichia coli* result in the production of insoluble proteins in both semi-pro-PM-I and its truncated form, and thus require protein refolding. Moreover, the yields of purified, soluble PM-I from all reported studies are very limited. Exclusion of truncated semi-pro-PM-I expression in *E. coli* C41(DE3) is herein reported. We also show that the low preparation yield of purified semi-pro-PM-I with autoprocessing ability is mainly a result of structural instability of the refolded enzyme in acidic conditions due to incomplete formation of disulfide linkages. Upon formation of at least one of the two natural disulfide bonds, nearly all of the refolded semi-pro-PM-I could be activated to its mature form. A significantly improved yield of 10 mg of semi-pro-PM-I per liter of culture, which resulted in 6–8 mg of the mature PM-I, was routinely obtained using this strategy.

## Introduction

Malaria is a serious public health challenge caused by *Plasmodium* parasites, annually resulting in 300–660 million clinical cases and approximately one million deaths [1], [2]. Among the five species of *Plasmodium* that infect humans, *P. falciparum* is the most virulent as it causes fatally severe anemia and cerebral malaria. Moreover, most of the available anti-malarial drugs have become ineffective due to the development of resistant parasites, highlighting the need to develop new and potent anti-malarials. Biochemical pathways of the parasites have been analyzed and several enzymes have been proposed as potential drug targets, although only a few have been validated [3].

A group of interesting targets belong to the hemoglobin-degrading pathway, which supplies amino acids for the growth and development of parasites. In the erythrocytic stage, several enzymes in the parasite food vacuole degrade human host hemoglobin. These include the aspartic proteases plasmepsins (PM) I, II, and IV, and the closely related histoaspartic protease (HAP); cysteine proteases falcipains-2 and 3; the metalloprotease falcilysin; dipeptidyl aminopeptidase-1; and possibly other aminopeptidases [4]. Hemoglobin degradation occurs in a semi-ordered process initiated by PM-I and II. These enzymes cleave a peptide bond between Phe33 and Leu34 at the hinge region of α-globin responsible for stabilizing the native hemoglobin tetramer. The cleavage unravels the protein, exposing it to further digestion. Moreover, peptide-like aspartic protease inhibitors such as SC-50083, Ro 40-4388, and “compound 7” are potent against PMs and the parasites at nanomolar to micromolar concentrations [5]–[7], suggesting the PMs are potential drug targets [8]. However, a study on disruption of genes encoding the vacuolar proteases shows that the doubling times of triple knock-out (PM-I, IV, and falcipain-2) parasites are longer than either PM or falcipain single knock-outs alone [9], raising a question about the validity of PMs as single drug targets. Nevertheless, the fact that the aspartic protease inhibitor pepstatin-A is more potent against the falcipain knock-out parasites than either wildtype or PM knock-outs [9] does not rule out the potentials of PM-inhibitors as part of combined anti-malarial drug therapy.

Purification of PMs from natural source is difficult and provides a very limited amount of protein, thus heterologous expression is an attractive alternative [10]. PM-II, IV, and HAP have been successfully recovered from inclusion bodies, whereas the preparation of PM-I is more challenging [11]–[15]. Expression of the full-length pro-PM-I yields a truncated product starting at Met73p (p indicates numbering in the pro-segment) without autoactivation activity, suggesting that the protein may fold incorrectly [12]. Preparation of the refolded semi-pro-PM-I with autoprocessing activity has been achieved with a construct starting at Met73p containing a Lys110p→Val mutation [6], but with a limited yield of 900 µg activatable semi-pro-PM-I from a 14-liter culture. Subsequently, the purification of a soluble thioredoxin–semi-pro-PM-I (starting at Lys77p) fusion protein has been developed, but the production yield of 62 µg/l culture is rather scanty [16]. The best preparation yield of 1.2–2.1 mg/l culture of a related semi-pro-PM-I construct starting at Ser76p with a Lys110p→Asn mutation has recently been reported [17]. The improved protein yield allows for the study of PM-I subsite specificity using peptide libraries, but remains marginal for studies demanding large amounts of pure PM-I enzyme including crystallization experiments. In order to cope with those studies, a more efficient method for PM-I production is necessary. Herein we report on the improved preparation of recombinant semi-pro-PM-I in *E. coli* that provides multi-milligrams of purified, activatable semi-pro-PM-I from 1-liter of culture without the need for a fusion-protein partner.

## Materials and Methods

### Construction of the Pro-PM-I Gene

**Figure 1:**
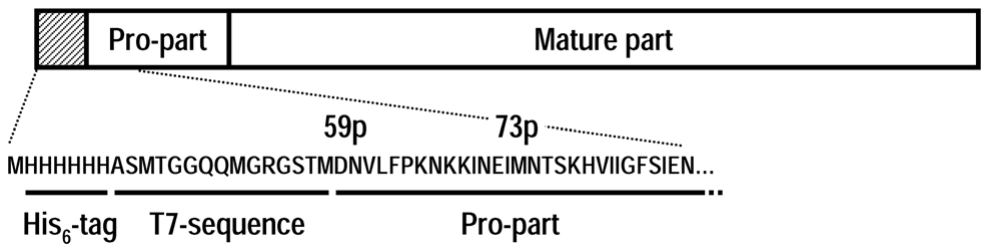
The recombinant pro-PM-I construct used in this study. The hashed box represents the His_6_-tag and T7 sequence located at the N-terminus, followed by the PM-I sequence starting at Asp59p. The construct used in most of other studies generally begins with Met73p.

Plasmid pET3a harboring semi-pro-PM-I gene was a gift from Dr. Richard P. Moon. The gene contains the last 63 pro-part residues (starting at Asp59p), followed by 331 mature-PM-I residues. A His_6_-tag was added to the N-terminus to facilitate purification (Figure 1), using the QuickChange mutagenesis kit (Stratagene) and oligonucleotide primers 5′-GAAGGAGATATACATATGCATCACCATCACCATCACGCTAGCATGACTGGTG-3′ and 5′-CACCAGTCATGCTAGCGTGATGGTGATGGTGATGCATATGTATATCTCCTTC-3′, containing the underlined inserts. After DNA sequence verification, the selected vector was subsequently transformed into *E. coli* expression hosts.

### Expression of PM-I Zymogen

**Figure 2:**
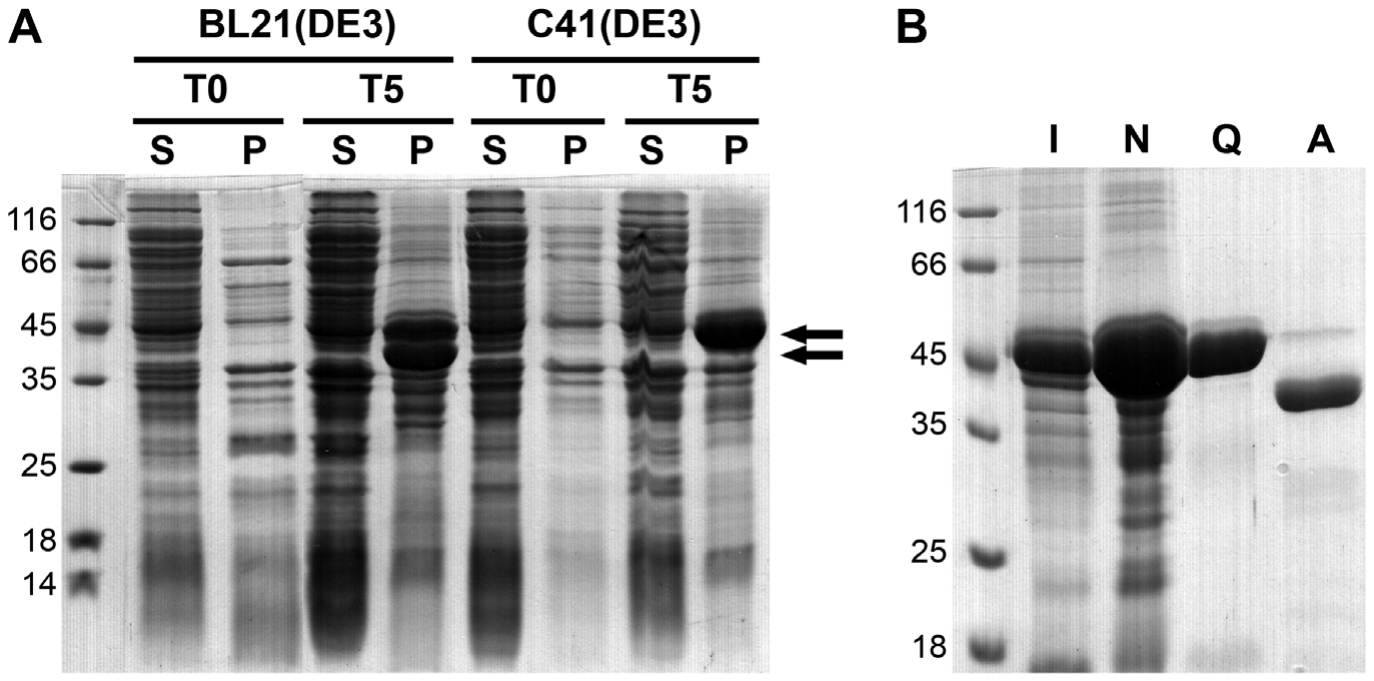
SDS-PAGE analysis showing the expression and purification of semi-pro-PM-I. (A) Expression of semi-pro-PM-I in BL21(DE3) pLysS and C41(DE3). Supernatants (S) and pellets (P) from lysed cells obtained before (T0) and after 5-hour induction (T5) are compared. Arrows indicate expression of the semi-pro-PM-I and its truncated product. (B) Samples obtained at various purification steps: I, urea-solubilized inclusion bodies; N, post Ni-column; Q, post Q-Sepharose; and A, activated PM-I after prolonged dialysis.

Two strains of *E. coli*, BL21(DE3) pLysS and C41(DE3) (Lucigen), were used in this study. For expression of recombinant proteins in BL21(DE3) pLysS, a single colony of the transformed cells was pre-cultured in LB medium supplemented with 100 µg/ml ampicillin and 35 µg/ml chloramphenicol overnight at 37°C followed by inoculation of a large-scale culture. Cells in 1-liter cultures were grown at 37°C until the optical density at 600 nm reached 0.5. Expression of the recombinant protein was then induced with 1 mM isopropyl β-D-1-thiogalactopyranoside (IPTG) at 37°C for 1, 3, and 5 h. Cells were harvested by centrifugation at 5700 g for 20 min at 4°C. For C41(DE3) cells, the growth and induction conditions were the same as those used for BL21(DE3) pLysS cells, except that only ampicillin selection was used. For SDS-PAGE analysis, aliquots of harvested cells before and after the induction, normalized based on cell density, were resuspended in buffer A (20 mM Tris HCl pH 8.0, 150 mM NaCl, and 5 mM dithiothreitol (DTT)) and lysed by sonication. The soluble components were separated from insoluble material by centrifugation at 10400 g for 10 min. The insoluble material was resuspended in equal volumes of buffer A and repelleted twice to remove any contaminating soluble proteins. Equal volumes of the supernatants and insoluble pellets were analyzed using 12.5% SDS-PAGE (Figure 2).

### Isolation and Solubilization of Inclusion Bodies

Harvested cells were resuspended in buffer A and sonicated on ice. Cell debris and inclusion bodies were pelleted by centrifugation at 10400 g for 10 min and washed with 1% CHAPS in buffer A twice, followed by 3 washes with buffer-A. For each washing step, the pellet was homogenized in the corresponding buffer and repelleted by centrifugation at 10400 g for 10 min. The washed pellet was then denatured in 20 mM Tris HCl pH 8.0, 150 mM NaCl, and 8 M urea, stirred at room temperature for 4 h, and dialyzed overnight against 10x volume of dialysis buffer (20 mM Tris HCl pH 8.0 and 6 M urea). Samples were filtered through 0.45-µM Durapore membrane (Merck Millipore) to remove any insoluble particles.

### Purification and Refolding of Denatured Semi-pro-PM-I

The denatured semi-pro-PM-I was purified using a Chelating Sepharose Fast Flow (GE Healthcare) column (Ø 16 mm, 30 ml) charged with nickel sulfate following the manufacturer’s protocol. After washing with 10x column volume of the dialysis buffer, the enzyme was eluted with 50 ml of a 0–500 mM imidazole gradient. Fractions containing the 45-kDa protein band were pooled and dialyzed against the dialysis buffer twice at 4°C. The denatured samples (3–5 mg/ml) were filtered through 0.45-µM Durapore membrane and then refolded using a ten-volume rapid dilution into 20 mM Tris HCl pH 8.0, 1 mM reduced glutathione (GSH), 0.1 mM oxidized glutathione (GSSG), and 10% glycerol, with stirring at 25°C for 10–50 h. Precipitates were removed by centrifugation for 10 min at 10400 g. The supernatant was filtered and loaded onto a Q-Sepharose Fast Flow (GE Healthcare) column (Ø 26 mm, 80 ml) pre-equilibrated with 20 mM Tris HCl pH 8.0 at 25°C. After washing with the same buffer, semi-pro-PM-I was eluted with the following NaCl gradients in 20 mM Tris HCl pH 8.0∶30 ml of 0–0.3 M, 20 ml of 0.3 M, 70 ml of 0.3–1 M, and 30 ml of 1 M. The eluted fractions were analyzed on SDS-PAGE to check for purity. Fractions containing high purity of semi-pro-PM-I were pooled and dialyzed against 20 mM Tris HCl pH 8.0 at 4°C for up to three days. Aliquots of the dialyzed sample were taken at 0, 3, 6, 9, 24, and 48 h for determination of free thiol groups and autoactivation ability. Concentrations of the purified semi-pro-PM-I were determined spectrophotometrically using the extinction coefficient at 280 nm of 4.506×10^4^ M^–1^ cm^–1^ as calculated using ProtParam [18].

### Determination of Free Thiol Groups

Free thiol groups were measured using Ellman’s reagent, 5,5′-dithiobis(2-nitrobenzoic acid) (DTNB) prepared in 0.1 M sodium phosphate pH 7.0. Samples were incubated at 25°C for 15 min and the absorption at 412 nm was recorded and used to calculate the molar concentration of free thiols from the extinction coefficient of reduced Ellman’s reagent of 1.415×10^4^ M^–1^ cm^–1^ [19].

### Activation of Recombinant Semi-pro-PM-I

The precursor was activated by addition of one-tenth volume of 250 mM sodium acetate pH 4.5. For the study of pH-dependent activation profile, sodium formate pH 3.5, sodium acetate pH 4.0 or 4.5, sodium citrate pH 5.0 or 5.5, or sodium phosphate pH 6.0 buffers at 250 mM were used. Reactions were incubated at 25°C for at least 2 h, and then stopped with the additional 0.15x volume of 2 M Tris HCl pH 8.8. Precipitated proteins were separated by centrifugation at 16060 g for 10 min. Completion of the activation was determined using 12.5% SDS-PAGE analysis.

### Assay of Enzyme Kinetics

The kinetic parameters of PM-I were assayed with the fluorogenic peptide DABCYL-Glu-Arg-Nle-Phe*-Leu-Ser-Phe-Pro-EDANS (Anaspec, * denotes the scissile bond) using a Cary Eclipse Fluorescence Spectrophotometer (Agilent Technologies) with excitation and emission wavelengths at 336 nm and 490 nm, respectively. The sequence of the peptide was based on the primary cleavage site of human hemoglobin by PM [20]. The appearance of the product was measured at 37°C for up to 600 s. Linear initial rates at different substrate concentrations were fitted to the Michaelis–Menten equation to determine the kinetic parameters. Reactions were performed in 100 mM sodium acetate pH 4.5 and started with the addition of 10 nM mature PM-I.

## Results

### Recombinant Expression of Semi-pro-PM-I

Preliminary expression studies of the semi-pro-PM-I in *E. coli* BL21(DE3) pLysS at 37°C showed two major protein bands expressed as inclusion bodies with the sizes around 45 and 40 kDa, the latter band presumably a truncated semi-pro-PM-I (Figure 2A). In contrast, only one major band with the expected size of the complete semi-pro-PM-I was observed in *E. coli* C41(DE3). Moreover, the use of the C41(DE3) expression host appeared to result in the production of a higher amount of semi-pro-PM-I in inclusion bodies when compared to BL21(DE3) pLysS (Figure 2A). Because of these two advantages the C41(DE3) strain was selected for large-scale production of the semi-pro-PM-I.

### Purification of Semi-pro-PM-I

**Figure 3:**
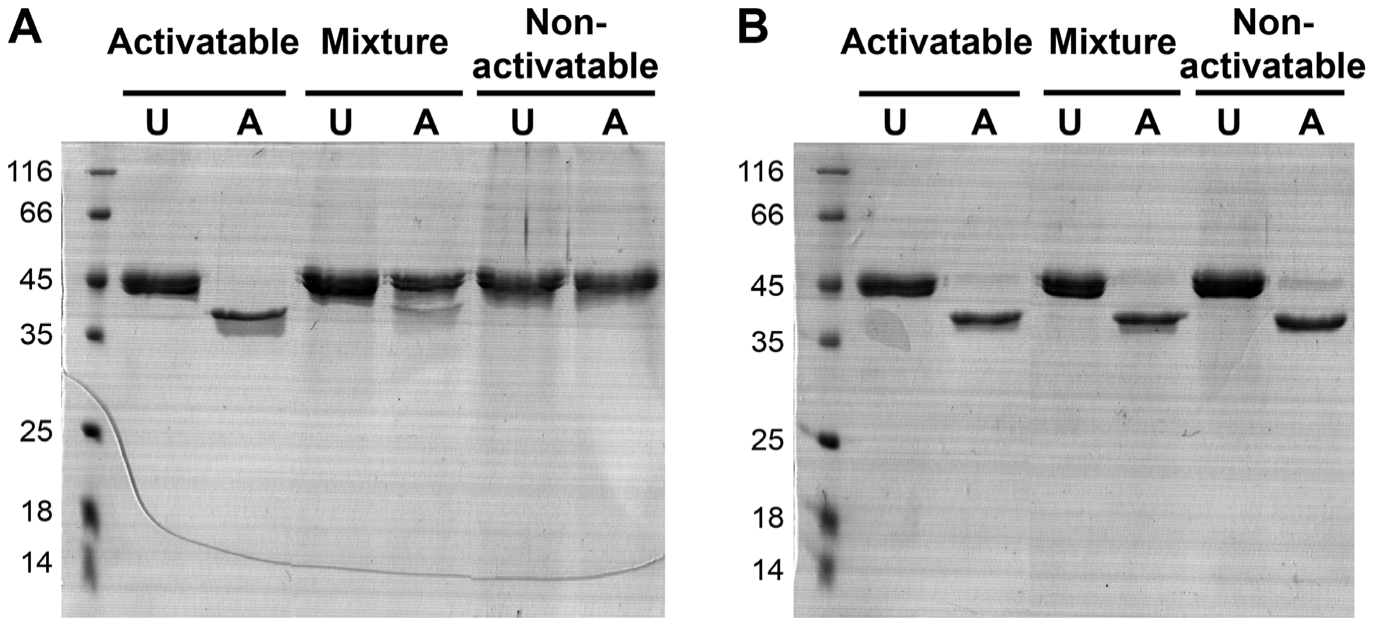
Autoprocessing abilities of purified semi-pro-PM-I fractions. Autoprocessing results of samples before (A) and after 3-day dialysis (B) were analyzed with SDS-PAGE. Three samples after Q-Sepharose chromatography were tested for autoactivation at pH 4.5: activatable and non-activatable pools, and a 1∶1 mixture. Equivalent volumes of soluble samples before (U) and after (A) 2-hour activation are compared.

The semi-pro-PM-I inclusion bodies expressed in C41(DE3) cells were washed and denatured with 8 M urea. The denatured samples were then dialyzed against 6 M urea and purified with a Ni-chelating column. The SDS-PAGE analysis revealed that the protein contaminants were not completely removed with this first column (Figure 2B). The fractions containing high amounts of semi-pro-PM-I were pooled, dialyzed, and refolded by rapid dilution. The refolded protein was further purified using a Q-Sepharose column with native conditions. A broad peak eluted at approximately 0.3 M NaCl contained a major protein band of 45-kDa that could be divided into two populations: activatable and non-activatable fractions. The latter form was observed in fractions at the tail of the peak. Both semi-pro-PM-I forms apparently were monomeric as evidenced by native-PAGE, in contrast to the broader peak of aggregated semi-pro-PM-I eluted with higher NaCl concentrations (data not shown). Instead of discarding this non-activatable form, we combined it with the activatable form to determine if activation could occur in *trans*. Both semi-pro-PM-I forms and a 1∶1 mixture were placed in three different bags for dialysis in the same container to ensure the same buffer compositions for subsequent autoactivation tests. After a prolonged dialysis (three days), all three samples were nearly completely activated (Figure 3), indicating that the activity in the non-activatable pool could be recovered in the absence of the activatable semi-pro-PM-I. This recovery of activity significantly improved the yield of purified semi-pro-PM-I.

### Determination of Free Thiol Groups

**Figure 4:**
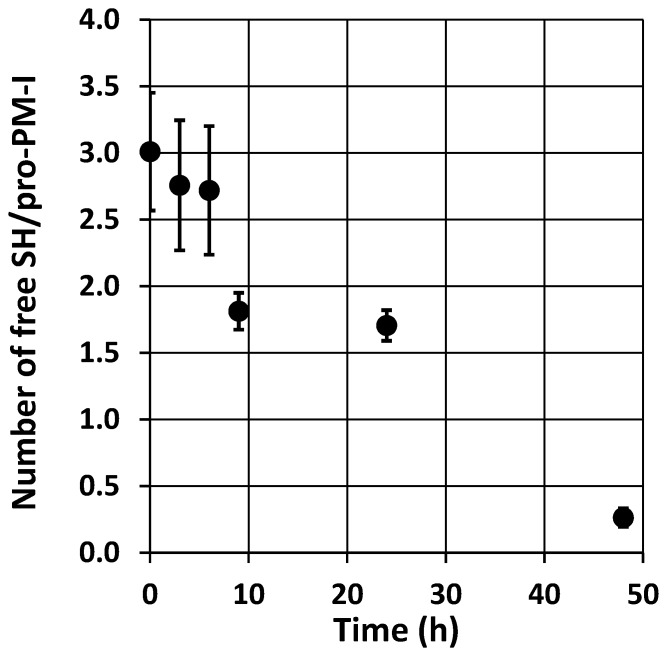
Determination of free thiols in semi-pro-PM-I samples during a prolonged dialysis. Average numbers of free sulfhydryl groups per semi-pro-PM-I molecule were determined in triplicate samples collected at 0, 3, 6, 9, 24, and 48 h. Error bars indicate ranges of determined values.

**Figure 5:**
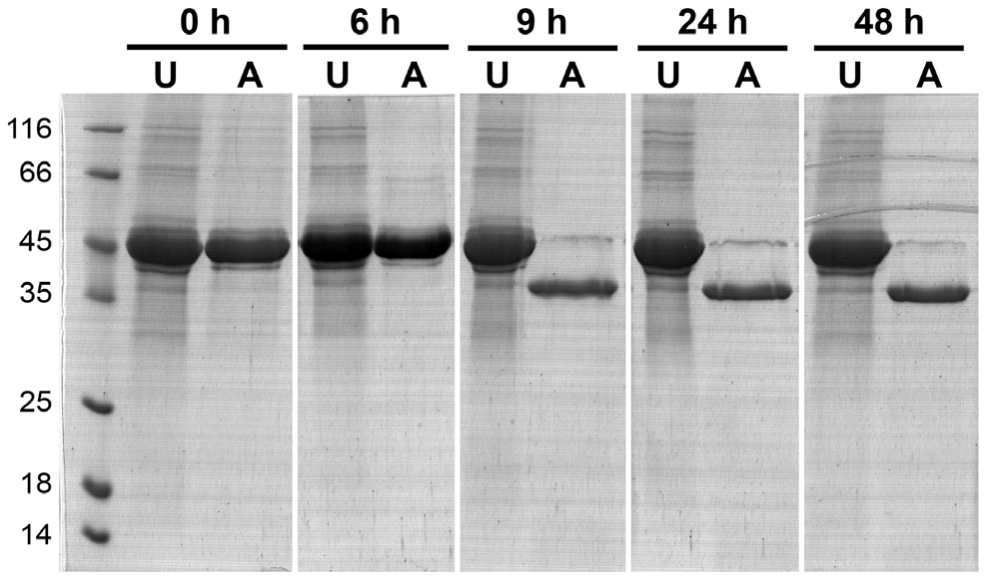
Autoprocessing abilities of semi-pro-PM-I samples during a prolonged dialysis. Autoactivation abilities of the samples from the same dialysis experiment as shown in Figure 4 at 0, 6, 9, 24, and 48 h were evaluated for protein band shift in SDS-PAGE from around 45-kDa unactivated samples (U) to 37-kDa mature PM-I (A).

Varying the compositions of refolding buffers demonstrated that reagents that modified thiol oxidation state, e.g. β-mercaptoethanol, DTT, GSH, and GSSG, affected the distribution ratios between the two populations. Compounds promoting disulfide reduction tended to increase the non-activatable proportion while GSSG had the opposite effect. The PM-I sequence contains four cysteine residues: Cys47, Cys52, Cys249, and Cys285, which are conserved among the PM enzymes. These equivalent residues in PM-II, IV, and HAP structures form two disulfide bonds (PDB: 1LS5 for PM-IV) [7], [21]. We hypothesized that the conversion of non-activatable semi-pro-PM-I to its activatable form upon the dialysis might be caused by air oxidation of the thiol groups to form equivalent disulfide bonds in the semi-pro-PM-I structure, which may stabilize the structure in the acidic-autoactivation condition. To test our hypothesis, the amount of free thiol groups was quantitated using the Ellman assay. After 9 h of dialysis, the average amount of 3.01±0.44 (mean and range) thiols per molecule of semi-pro-PM-I dropped to 1.81±0.14, and then to 0.26±0.07 after 48 h (Figure 4). The zymogen dialyzed for at least 9 h showed good activation ability (Figure 5).

### Biochemical Characteristics of Recombinant PM-I

In an optimized preparation, after an overnight refolding and Q-Sepharose purification, the fractions with high purity of semi-pro-PM-I were pooled and dialyzed overnight at 4°C. This procedure yielded about 10 mg of activatable precursor per one liter of culture with 95% homogeneity as judged with SDS-PAGE (Figure 2B).

The purified semi-pro-PM-I was incubated at 37°C overnight in different buffers over a pH range of 3.5–6.0. The protein underwent activation at pH below 5.0, with the pH optimum at 4.5 as revealed by a complete band shift from 45 kDa to 37 kDa (data not shown). In addition, mature PM-I was produced with the standard protocol and its kinetic parameters were measured at pH 4.5. The *K_M_* and *k_cat_* values are shown in Table 1 and compared with similar parameters from other studies.

**Table 1:** Comparison of kinetic parameters of recombinant PM-I from various studies.

*K_M_* (µM)	*k_cat_* (s^−1^)	*k_cat_*/*K_M_* (mM^–1^s^–1^)	Reference
0.092±0.004	0.344±0.050	3736	This study^a^
0.917±0.144	0.473±0.172	528	[24] b
0.49±0.12^c^	2.3^c^	4700^c^	[12] b
13±1^d^	0.24^d^	20^d^	[12] b
8^c^	16^c^	2000^c^	[10] e
10	2.1	210	[10] e
8.6±0.2	2.6±0.1	302	[17] e
10	1.3	120	[6] e

a Determined with fluorogenic peptide DABCYL-Glu-Arg-Nle-Phe*Leu-Ser-Phe-Pro-EDANS at pH 4.5, and reported as mean ± SD of triplicate measurement.

b Determined with fluorogenic peptide DABCYL-GABA-Glu-Arg-Met-Phe*Leu-Ser-Phe-Pro-GABA-EDANS at pH 5.0.

c Purified, naturally occurring PM-I.

d No conversion to the mature PM-I is observed by SDS-PAGE.

e Determined with chromogenic peptide Leu-Glu-Arg-Ile-Phe*Nph-Ser-Phe.

## Discussion

The *P. falciparum* food vacuole contains at least three PMs and HAP enzymes that have been proposed as new drug targets against human malaria [4], [8]. While reports on structures and functions of PMs II and IV, and HAP have been made available, those of PM-I are quite limited mainly due to the low yield of active enzyme that can be prepared from heterologous expression systems. Previously, the full-length enzyme was cloned and expressed in *E. coli* in a truncated form starting at Met73p [12]. In this study, a semi-pro-PM-I construct starting at Asp59p, which is the residue located right after the putative trans-membrane region, was used (Figure 1). Expression of this gene construct in *E. coli* BL21(DE3) pLysS resulted in two forms: the expected semi-pro-PM-I and its truncated product, possibly due to alternate translation site or proteolytic activity. Substitution of Met73p by Leu, which is the equivalent amino acid in PM-II, did not prevent the expression of the truncated form (our unpublished data). Since this was accomplished by replacement of the AUG (Met) codon with TTA (Leu) codon as observed in the PM-II gene sequence, and not the TTG (Leu) codon which could serve as an alternative initiation codon [22], a possibility that Met73p might act as an alternate initiation site could be reasonably ruled out. In contrast, expression of the truncated form was significantly reduced in *E. coli* C41(DE3). This cell type is a mutant of *E. coli* BL21(DE3) that can survive the toxic effects due to expression of various proteins [23]. Although the mutation has not been fully characterized, the use of this cell type for expression of the semi-pro-PM-I suggested that expression of the truncated form might be prevented by suppression of proteolysis at the site. To our knowledge, this is the first evidence demonstrating the advantage of the C41(DE3) strain in reducing truncated protein expression.

Currently, the reported refolding methods of semi-pro-PM-I employ either a rapid or step dilution of the urea denaturant [6], [17], [24]. The efficiency of such refolding is generally determined from the autoprocessing ability of the refolded zymogen, and the recovery yields are quite low. In this study, the protein was refolded by rapid dilution of urea, followed with Q-Sepharose chromatographic purification. The fractions containing purified semi-pro-PM-I were pooled and dialyzed against 20 mM Tris HCl pH 8.0 for up to three days, which could convert the non-activatable semi-pro-PM-I pool to a usable form. The experiments with a mixture of the two semi-pro-PM-I pools neither supported nor ruled out the *trans* activation mechanism, as such conversion was also possible in the absence of the activatable population (Figure 3). However, the activation of both pools significantly improved the yield of usable semi-pro-PM-I as a large proportion of the non-activatable form could be recovered.

Using the Ellman assay to monitor the number of free thiol groups in the dialyzed semi-pro-PM-I, we found a correlation between the formation of disulfide bonds and the autoprocessing ability. Samples dialyzed up to 6 h contained an average number of free sulfhydryl groups per semi-pro-PM-I molecule of 2.72 or higher (out of four Cys residues) and could not be activated, while those dialyzed 9 h or longer processed readily (Figures 4, 5). The activatable semi-pro-PM-I had an average number of free thiol groups per molecule below two, indicating that there was at least one disulfide bond formed. Together with the observed effects of chemicals that modified thiol oxidation states on autoprocessing activity of semi-pro-PM-I, it appears that disulfide-bond formation was essential for activation ability of the zymogen, and that refolding of semi-pro-PM-I was not directly coupled with its processing property. Indeed, the refolding of semi-pro-PM-I via rapid dilution of urea would be very efficient, producing large amount of the zymogen in its native or near-native structures. These forms of semi-pro-PM-I were unstable in acidic environments and predominantly present as aggregated or precipitated proteins (data not shown). However when at least one disulfide bond was formed, in this case likely by air oxidation occurring upon the prolonged dialysis, the proteins were stabilized as activatable monomers. This observation suggests a role for the disulfide bonds of PM-I precursor on its structural stabilization in acidic environments, which are found in *in vitro* experiments of autoprocessing and activity assays, as well as at its subcellular site in *P. falciparum* food vacuoles. Similar studies on stabilization of tertiary structure by disulfide bonds upon protein folding have been reported, including the oxidative refolding of prochymosin [25]. However, for prochymosin refolding, the disulfide bonds may form at even earlier state and can facilitate the formation of its native tertiary structure. In addition, a significantly increased preparation yield of *P. falciparum* PM-II, which has two similar disulfide linkages in its native structure, has also been observed with a prolonged incubation prior to autoactivation (data not shown).

The kinetic parameters of PM-I prepared in this study cannot be directly compared with those characterized earlier as different substrates and assay conditions have been used (Table 1). Comparing with the chromogenic peptide substrate, the larger sizes of the fluorogenic peptides and the presence of aromatic fluorescent resonance energy transfer (FRET) groups at the equivalent positions of hydrophobic or mildly polar amino acids in the α-globin sequence around the initial cleavage site (ALERMF*LSFPTT, with the underlined amino acids representing those equivalent positions) make them more resemble the native globin substrate. Liu *et al.* have demonstrated that PM-I prefers nonpolar or uncharged, polar amino acids for its P3–P3’ subsite specificity and that there are still spaces flanking the peptidomimetic inhibitors, which are modeled into the PM-I binding groove based on the inhibitor-bound PM-II structure [17]. These possibly allow the FRET peptides to form greater numbers of interactions with the relatively hydrophobic active site of PM-I, resulting in lower *K_M_* values than those of the shorter chromogenic peptide in general (Table 1). The question whether these parameters better represent those of the physiological substrate remains to be proven with more studies on PM-I kinetics towards the globin substrate or peptidomimetic inhibitors, and perhaps three-dimensional structures of these complexes. Comparing with the studies in which a fluorogenic substrate of similar sequence is utilized, the catalytic rate constant reported herein is similar to that described by Siripurkpong *et al.* [24] and the catalytic efficiency *k_cat_/K_M_* is relatively close to that reported by Luker *et al.* [12] (Table 1). The *K_M_* reported in this work is, however, significantly lower, which is probably due to the different substrate sequences and assay conditions. The ionization of acidic amino acids would be more substantially affected by the pH difference in those assay conditions (4.5 vs. 5.0) as their pKa’s are closer to these values than other types of amino acids on both the enzyme and substrate. Theoretically, there should be approximately 3-fold increase in the ratios of their uncharged, carboxylic acid versus carboxylate anion forms if the pH is changed from 5.0 to 4.5. It is conceivable that reduction in the average charge of Glu residue at P4 position on the FRET peptide at pH 4.5 may contribute to the lower *K_M_* observed in this report. In addition, Luker *et al.* have shown significantly different kinetic parameters between native PM-I and a recombinant pro-PM-I that was truncated at Met73p in the preparation (Table 1) [12]. The latter produces no observable conversion to the mature PM-I in SDS-PAGE, while the semi-pro-PM-I prepared in this study is readily activatable. Despite the minor differences in terms of substrates and assay conditions used, the PM-I reported herein demonstrates even greater similarity of kinetic parameters to those of the native PM-I than those of the truncated, recombinant pro-PM-I reported in the same study [12], suggesting that the structure of this semi-pro-PM-I more resemble the native protein. However, the question whether such difference in *K_M_* values is also a result of different enzyme stabilities or other factors should be investigated further using the same type of substrate and assay conditions.

Recent studies have shown that the soluble PM-I enzyme can be produced using the thioredoxin fusion technology, and crystals of the mature enzyme can be produced from this fusion protein construct [16], [26]. Because the PM-I construct contains thioredoxin at the N-terminus, its utility in structural studies requires removal of the fusion partner as the fused thioredoxin domain may interfere with the structure or other properties of the zymogen. The reported yield of the soluble fusion enzyme is 62 µg from one-liter of culture [16] and another study has reported a yield of 1.2–2.1 mg PM-I precursor per liter of culture, which is a borderline amount for structural investigations [17]. Although the structural report does not state the yield of PM-I production, it clearly shows that the authors achieved it with difficulty of limited supply or stability of the purified protein as seen from the experimental design and relatively poor diffraction statistics despite the use of strong synchrotron radiation [26]. In this study, 10 mg of the purified semi-pro-PM-I, which resulted in 6–8 mg of the mature PM-I, could be routinely produced from one liter of culture, providing a significantly improved yield of usable recombinant PM-I protein.

## Conclusions

We have devised a simple and efficient protocol for the refolding of recombinant semi-pro-PM-I from *E. coli* inclusion bodies into stable zymogen forms, which can be activated *in vitro* to yield large amount of the biologically active PM-I. Furthermore, our findings also suggest that at least one disulfide bond is required for stabilization of the functional PM-I structure at its optimal, acidic condition. Such high preparation yield and stability of PM-I obtained with this protocol should enable further studies on its processing, biological functions, inhibition, as well as structures. Moreover, the approach we present here may be applied to refolding of other PMs and other aspartic proteases as these enzymes are most efficient in the acidic pH range.
